# Ecological and Parasitological Study on *Cerithidea cingulata* (Gastropoda) in Hormoz Strait Littoral, South of Iran

**Published:** 2018

**Authors:** Mehdi KALAT-MEIMARI, Jebreil SHAMSEDDIN, Abdoreza SALAHI-MOGHADDAM

**Affiliations:** 1. Dept. of Medical Parasitology, Faculty of Medicine, Hormozgan University of Medical Sciences, Bandar Abbas, Iran; 2. Infectious and Tropical Diseases Research Center, Hormozgan University of Medical Sciences, Bandar Abbas, Iran

**Keywords:** *Cerithidea cingulata*, Parasitology, Ecology, Malacology, Iran

## Abstract

**Background:**

*“Cerithidea cingulata”* is reported from south of Iran, may act as intermediate host of *Heterophyes heterophyes* and cercaria dermatitis. As parasitological aspects of this brackish snail were not studied in Iran, this study was conducted in 2016, in Hormoz Strait, south of Iran.

**Methods:**

Totally 402 snails were collected from 36 locations of three main regions in Hormozgan Province, Iran in 2015. In each location, one square meter was checked, and snails were collected for parasitological study. Snails were crashed and cercariae were studied using light microscope, in some cases natural red staining was used for better resolution.

**Results:**

Mean length of snails was about 20.33 mm, width 5.57 mm. The aperture length was 5.10 and spire was 15.22. Important founded cercaria was cysticercus cercariae, echinostoma cercaria, furcocercus cercaria, furcocystocercous cercaria, gastrostomy cercaria, gymnocephalus cercaria, monostome cercaria, pelurolophocercus cercaria and xiphidiocercaria.

**Conclusion:**

The presence of this snail is reported from Persian Gulf to western Pacific in China. Our study showed a pattern of infection in local area and was compatible with other studies. Importance of *C. cingulata* as intermediate host of some medically important disease should be considered and other complementary molecular studies for exact identification of cercaria are necessary.

## Introduction

*Cerithidea cingulata”* is known as “*Pirenella cingulata”*, is a proso-branch, operculated brackish water snail. According to US National Center for Biotechnology Information (NCBI), taxonomy of *Cerithidea* may consider as follows (1); Class; Gastropoda, Oder; Sorbeoconcha, Superfamily; Cerithioidea, Family; Potamididae, Genus; *Cerithidea*.

Hormoz Strait is an important area in south of Iran, where the Persian Gulf (2) is separated from Oman Sea. Ecology of this area is affected by three zoogeographic zones, Sahero-Arabian in south Palearctic in north and Indo-Malayan in east (3). Epidemiological condition of disease in north of Persian Gulf and Oman sea is almost different from other parts of Iran. While most parts of Iran is known as Palearctic zoogeographical area, south of Iran may consider in Afrotropical-like and Indomalayan-like subzones. Some diseases like Schistosomiasis, Dracunculiasis (4) and Heterophyasis (5) were reported from this area and not from northern parts. Combination of different climatologic and zoogeographic realm and zones in Iran produce a complex epidemiological condition.

Brackish water or sea snails have less medically importance but “*Pirenella conica”* is known as intermediate host of *“Heterophyes heterophyes”* in some parts of the world (6). *“C. cingulata”* was reported from south of Iran (7) in 2011 for the first time (8), however, its parasitological importance as an intermediate host is not well studied in Iran.

For better understanding the role of *“C. cingulata”* in ecology and epidemiology of probable disease in specific local condition, this study was conducted in 2016 in Hormoz Strait, south of Iran.

## Materials and Methods

### Study area

**Fig. 1:**
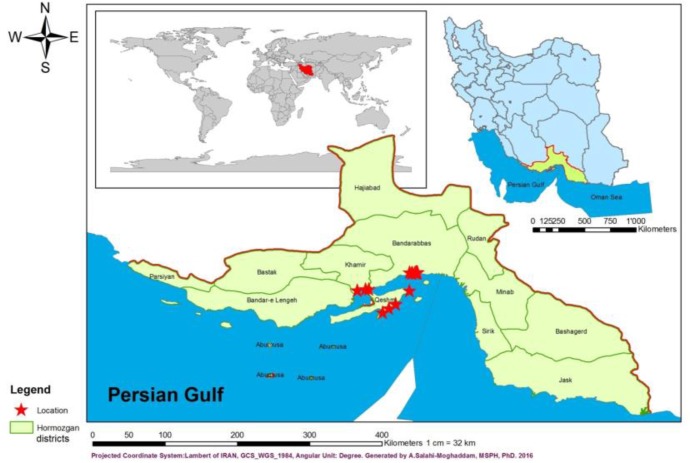
Hormozgan Province, Hormoz Strait and studies area

Totally, 402 snails were collected from 36 locations of three main regions in Hormozgan Province, adjust to Hormoz Strait, including “Khamir”, “Bandar-Abbas” and “Qeshm” districts in 2016 (Fig. 1).

In the study area, 36 locations were considered as suitable niche for snail colonies while 33 were success and 3 locations were empty. In each location, a 1x1 meter area was isolated by four 1 m wooden scale. Ecological condition of water was checked using portable multimeter (pH, EC, TDS, Salt, and thermometer) Model TS7200 MIC company. A portable oxygen meter (AZ Model 8403) was used for measuring dissolved oxygen. Ecological variable measurements were, Water temperature (Celsius Degrees; C), pH, Total Dissolved Salts (TDS in ppm), Oxygen (ppm), considering equal amount of Oxygen and Dissolved Salt, these variables were excluded.

Snails were transmitted to Hormozgan University of Medical Sciences, General Laboratory and were checked using crash method (9). Some suitable samples were stained using lactophenol methylene blue and natural-red and diagnosed by light microscopy. Cercaria density in each snail was recognized as Light, Moderate, Heavy and Very heavy. From some suitable samples, film and photography were taken and taken. We used a Neubauer chamber scale for calibrating microscope and measuring the size of cercaria in this study. Measurement was done using Image J 1.5i, National Institutes of Health-USA.

Results were arranged in SPSS 20, (IBM Corp Released 2011, IBM SPSS Statistics for Windows, Ver. 20 Armonk, NY: IBM Corp) and ArcGIS Desktop for Windows 9.3, (ESRI 2008, Redlands, CA, Environmental System Research Institute) for further analysis and mapping.

## Results

### Ecology and Conchology of C. cingulata

**Fig. 2:**
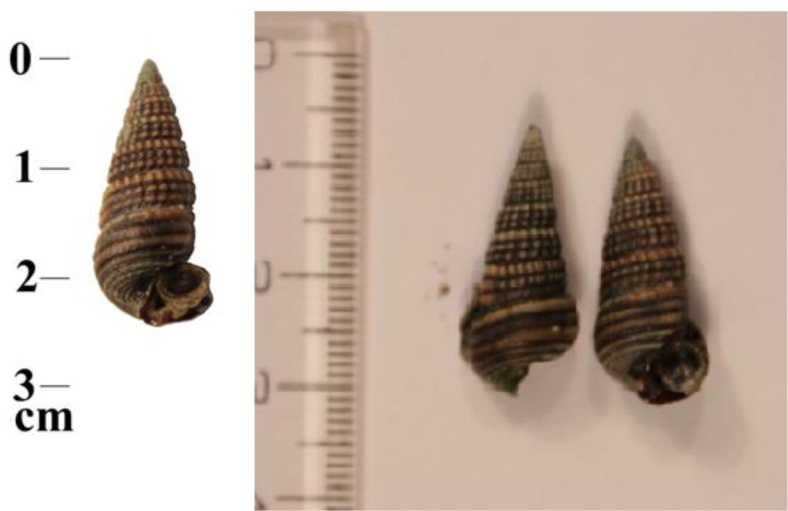
Cerithidea cingulata

Neglecting full grown snails or not, mean length of snails was 20.33 ± 7.85 mm, with mean width of 5.57 ± 0.67 mm. The aperture length was 5.10 ± 0.65 mm spire was 15.22 ± 5.02; Spire/Aperture rate was ∼3/1. Full grown snails had 6 to 7 brownish tuberculated whorls with semi deteriorated apex. Fig. 2 shows a sample of these snails.

Snails were collected from suitable niche in Hormoz Strait shore. Concerning low slope of shore, these samples were not adjusted to the water, interestingly bigger colonies had more distance from water in comparison with colonies in depth of shore; while 2.5% of samples were exactly in the water, 55.3% had considerable distance from water.

**Fig. 3:**
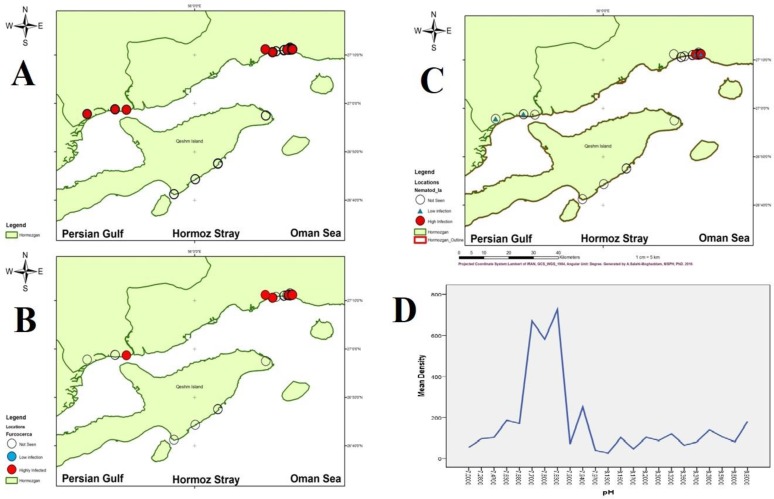
Ecological results of *Cerithidea cingulata* in Hormozgan Province, Southern Iran A: Distribution of infected snails in Persian Gulf littoral B: Distribution of furcocercaria infection C: Distributional map of nematode larvae D: Frequency of snail population in different pH

The average of water pH in the adjusted colonies was 8.06 ± 0.4, from minimum 7 to maximum 8.9. ANOVA analysis between grouped density and pH show statistical correlation (*P*=0.001) (Fig. 3- Part D).

The water pool had temperature from 22 to 39.5 with average of 34.00 ± 4.16. Mean of snails in one square meter was 155.42 with median 102, from minimum 10 to maximum 725.

### Parasitology

**Fig. 4:**
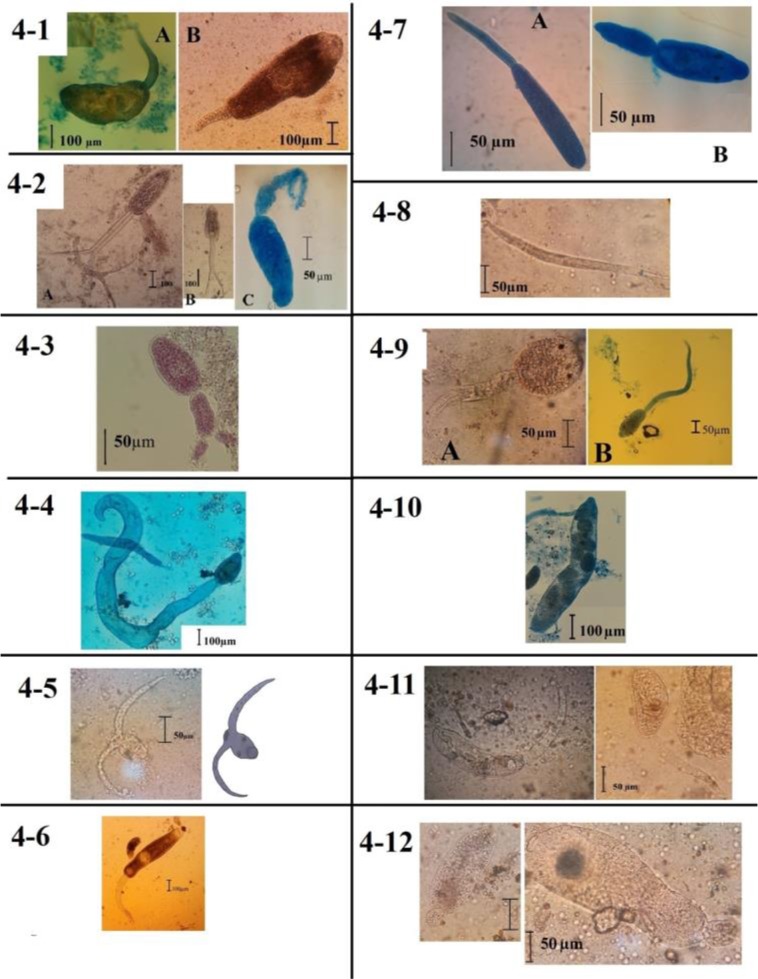
Different type of cercaria found in *Cerithidea cingulata* 4-1: Two samples of echinostoma cercaria. 4-1-A: Long tail cercaria. 4-1-B: Short tail cercaria. 4-2: Three types of furcocercus cercaria; A: large size classic furcocercaria, B: Small size classic furcocercaria, C: Spot eye furcocercaria 4-3: Furcocystocercus cercareae 4-4: Cystocercous cercariae 4-5: A gastrostom cercaria found around Hormoz Strait 4-6: Sample of gymnocephalus found in Hormoz Strait 4-7: Comparing two monostum cercaria found in this study 4-8: A nematode larvae 4-9: Pleurolophocercus cercaria 4-10: Sporocyst found in this study 4-11: Xiphidiocercaria found in *Cerithidea cingulata* 4-12: Cercarium with and without rostellum like organ

From 402 snails collected, 293 (72.9%) were not infected and 113 (27.9%) were infected to variety of cercaria, redi, sporocyst or nematode larvae. Fig. 4 shows distribution of infected snails in Persian Gulf Littoral. Table 1 shows biologic agents found in *C. cingulata*.

**Table 1:** Biological agents found in *Cerithidea cingulata* in Hormoz Strait

	***Biologic Agent***	***Sub type***	***Snail Count (N)***	***Percent (%) of snail***
1	Echinostome cercaria	2	11	2.73
2	Furcocercus cercaria	3	40	9.95
3	Furcocystocercous cercaria	1	38	9.5
4	Cystocercous cercariae	1	1	0.24
5	Gastrostome cercaria	1	1	0.24
6	Gymnocephalus cercaria	1	6	1.49
7	Monostomum cercaria	2	12	2.98
8	Nematode larvae	1	31	7.71
9	Pelurolophocercus cercaria	2	12	2.98
10	Redi and sporocyst	1	77	19.15
11	Xiphidiocercaria	1	4	0.99
12	Undefined Cases – Cercarium – Damaged cercaria	-	2	0.49

1- echinostoma cercaria

This cercaria was found in 11 snails. It may be considerd in two categories, based on size of the tail. About 2.73% of snails were infected to this kind of cercaria in which 1% had moderate and 1.7% had heavy infection. Two subtype echinostome cercaria may be considerd as follows:

The first was short tail cercaria with 600–610 μm body and 180 – 190 μm tail. (Fig 4-1-B)

The second echinostoma cercaria with long tail; in this case body has 410–420 μm with 330–310 μm. (Fig 4-1-A). Fig. 4-1 shows two samples of this cercaria type.

### Furcocercus cercaria

This cercaria was found in 40 snails and with 9.95%. Four different types of furcocercaria were seen in this study, including classic featured furcocercaria in two different sizes, with blunt shaped tale and with eyespot. 9.95% of snails have infected to this cercaria it was the most pollutant cercaria in the study areas. Eight percent of snails were highly infected to this cercaria. Figure 4-2 shows three sub-types of furcocercaria (Fig. 4-2-A to 4-2-C).

Furcocercaria in bigger size had a body with 360–370 micrometer the tail was 360–370 μm with two typical branches with 300–310 μm (Fig. 4-2-A).

The smaller typical furcocercaria was 200–210 μm in body with 190–200 μm tail and two branches with 180–185 μm (Fig. 4-2-B).

Spot eye furcocercaria had a body with 210 – 215 μm and a tail with 155–165 μm length and two branches with 95–100 μm length (Fig. 4-2-C).

Furcocystocercous cercaria was found in 9% of snails. Cercariae had body with 90–100 μm. the tail was about 65 μm and unlike others very oval shaped blunted tail end with two small blunted branches about 35 μm (Fig. 4-3).

Snails with infection with this cercaria were distributed around Bandar-Abbas and Bandar-Pol. Figure 3-B shows map of infected snail distribution.

### Cystocercous cercariae

Only one snail was moderately infected to this kind of cercaria. This cercaria had 240 μm length for body and 1900 to 2000 μm for tail. Eyespot was presented (Fig. 4-4).

### Gastrostomy cercariae

The only infected snail was slightly infected. Body of this cercaria was 65–75 μm with two 150–170 μm separated tail (Fig. 4-5).

### Gymnocephalus cercariae

There were 6 snails moderately infected to this cercaria. Hydrocephalus with long shaped morphology had a body about 690–700 with a tail with about 510–520μm (Fig. 4-6).

### Monostomun

Two sub-types of cercaria with one sucker were found in *C. cingulata* around Hormoz Strait, one smaller with eye spots and the second larger without eyespot.

Monostom cercaria with eyespot; from 12 infected snails 11 had moderate infection and one snail had very heavy infection to this cercaria. Size of body was about 155 with tail of 106 micrometer.

Monostome Cercaria with eyespot; Body 180–190 and tail 130–140.

Figure 4-7 shows two above mentioned sub-types of monostome cercaria.

### Nematode Larvae

This study shows infection of 12 snails to Nematode larvae. Mean size of these larvae was 350–370 μm in length. Fig. 4-8 shows distributional map of infected snails which are focused around Bandar-Abbas.

### Pelurolophocercus cercaria

Fifteen snails were infected with pelurolophocercus cercaria. Concerning morphological criteria, two morphological variations, were seen. The first cercaria had a round body 125 μm length x 113 μm width; its tail has 245 μm lengths (Fig. 4-9-A). Second pleurocercus cercaria had 160 μm length body and 426 μm tail (Fig. 4-9-B). In both cases, fin fold is not well projected in photography. Distribution of snails, infected with pleurolophocercus is similar to furcocercaria.

### Redi and Sporocyst

This study showed 77 snails were infected to different redi and/or sporocyst of trematoda. Typical size of these forms was 630–650 μm × 115–120 μm. Figure 4-10 shows a sporocyst.

### Xiphidiocercaria

Four snails infected with xiphidiocercaria were found. Measuring cercaria had results of cercaria with dominant body size about 163 and tail 196 micrometer. Figure 4-11 shows two xiphidiocercaria.

### Cercarium and Undiagnosed Cases

In addition to many crashed cercaria, segmented parts like tails or bodies which may not be identified, one case of micro cercaria and a case of unknown cercarium-like cercaria were seen, which had a rostellum like organ (Fig. 4-12).

## Discussion

The first available document about presence of *C. cingulata* in Iran refers to 2011(10) and this is the first and only study on ecology and parasitological aspects of *C. cingulata* in Iran. Presence of this snail is reported from Persian Gulf (11) to western Pacific in China (12). Another species of *Cerithidea* found in East Africa, where mangrove tree trunk may be found (13). Although Mangrove Forest is very close to our study area, there was not exactly in our sampling zone. Our findings on prevalence of different types of cercaria are compatible with studies of Nomchote in Thailand (10).

Our study shows infection of snails was not distributed normally; i.e. there were two high infected zones around Bandar Abbas and in the second rank Bandar-Khamir. While there was not any infected snail in the southern shore of Qeshm island, adjust to main areas of Hormoz Strait, where seagulls are more prevalent, most infected area with highest infection rate of snails was around the Bandar Abbas, capital of the province, with highest population density in the area. Source of snail infections was creek drainage coming from the land and seagull have less importance. The most prevalent cercaria in this study was furcocercaria.

Molecular studies in near area shows that it may act as Cercaria dermatitis agent (14) and is parallel to our findings on furcocercus cercaria.

## Conclusion

Freshwater snails are main intermediate hosts of trematoda and brackish water snails are have not any importance in public health but this study which is first study on brackish water snail shows probable importance of this snails in public health as probable intermediate host of *H. heterophyes*, concerning the reported pelurolophocercus cercariae. Complementary molecular studies on these cercariae and exact identification may be conducted by further research.
